# Slum compassionate community: expanding access to palliative care in Brazil

**DOI:** 10.1590/1980-220X-REEUSP-2022-0432en

**Published:** 2023-09-01

**Authors:** Maria Gefé da Rosa Mesquita, Alexandre Ernesto Silva, Lívia Pereira Coelho, Matheus Rodrigues Martins, Marcela Teixeira de Souza, Liana Amorim Corrêa Trotte

**Affiliations:** 1 Universidade Federal do Rio de Janeiro, Escola de Enfermagem Anna Nery, Departamento de Metodologia de Enfermagem, Rio de Janeiro, RJ, Brazil.; 2 Universidade Federal de São João del-Rei, Departamento de Enfermagem Fundamental, Divinópolis, MG, Brazil.; 3 Programa de Atenção Domiciliar ao Idoso, Rio de Janeiro, RJ, Brazil.; 4 Universidade Federal de Minas Gerais, Escola de Enfermagem, Programa de Pós-Graduação em Enfermagem, Belo Horizonte, MG, Brazil.; 5 Universidade Federal do Rio de Janeiro, Escola de Enfermagem Anna Nery, Programa de Pós-Graduação em Enfermagem, Rio de Janeiro, RJ, Brazil.

**Keywords:** Palliative Car, Vulnerable Population, House Call, Health Personne, Healthcare Model, Cuidados Paliativos, Poblaciones Vulnerables, Visita Domiciliaria, Personal de Salud, Modelos de Atención de Salud, Cuidados Paliativos, Populações Vulneráveis, Visita Domiciliar, Pessoal de Saúde, Modelos de Assistência à Saúde

## Abstract

**Objective:**

To describe the implementation of a compassionate community in Rocinha and Vidigal slums, located in the city of Rio de Janeiro.

**Method:**

Report on the experience of implementing a Compassionate Community based on the World Health Organization conceptual bases, supported by university extension guidelines.

**Results:**

Initially, local leaders and residents were recruited and trained in palliative care. Subsequently, health professionals from different specialties engaged in the project through volunteering. Home visits were instituted in the form of interconsultation and “sponsorships” by residents and health professionals to people in palliative care and family members. Finally, the health care network in the territory was integrated in order to recognize the project as a support network.

**Conclusion:**

We highlight the experience as living work in health, which involves relationships and creative processes, which mobilize structured technical knowledge and relationships between people and soft-hard and soft technologies, making it possible to recognize powers in the territory.

## RESUMO

**Objetivo:**

Descrever a implementação de uma comunidade compassiva nas favelas da Rocinha e Vidigal, localizadas na cidade do Rio de Janeiro.

**Método:**

Relato da experiência da implementação de uma Comunidade Compassiva a partir das bases conceituais da Organização Mundial da Saúde, amparada pelas diretrizes da extensão universitária.

**Resultados:**

Inicialmente, lideranças locais e moradores foram recrutados e receberam treinamento sobre cuidados paliativos. Posteriormente, profissionais de saúde de diferentes especialidades engajaram-se no projeto por meio da prática do voluntariado. Foram instituídas visitas domiciliares na modalidade interconsulta e “apadrinhamentos” por moradores e profissionais de saúde às pessoas em cuidados paliativos e familiares. Por fim, a Rede de Atenção à Saúde do território foi integrada de forma a reconhecer o projeto como rede de apoio.

**Conclusão:**

Destacamos a experiência como trabalho vivo em saúde, que envolve relações e processos criativos, os quais mobilizam o saber técnico estruturado e as relações entre as pessoas e as tecnologias leve-duras e leves, tornando factível o reconhecimento de potências no território.

## RESUMEN

**Objetivo:**

Describir la implementación de una comunidad compasiva en las favelas de Rocinha y Vidigal, ubicadas en la ciudad de Río de Janeiro.

**Método:**

Relato de la experiencia de implementación de una Comunidad Compasiva a partir de las bases conceptuales de la Organización Mundial de la Salud, sustentada en lineamientos de extensión universitaria.

**Resultados:**

Inicialmente, se reclutaron y capacitaron a líderes locales y residentes en cuidados paliativos. Posteriormente, profesionales de la salud de diferentes especialidades se involucraron en el proyecto a través del voluntariado. Se instituyeron visitas domiciliarias en la modalidad de interconsulta y “patrocinios” por parte de residentes y profesionales de salud a personas en cuidados paliativos y familiares. Finalmente, se integró la Red de Atención a la Salud del territorio para reconocer el proyecto como una red de apoyo.

**Conclusión:**

Destacamos la experiencia como trabajo vivo en salud, que involucra relaciones y procesos creativos, que movilizan saberes técnicos estructurados y relaciones entre personas y tecnologías ligeras-duras y ligeras, posibilitando el reconocimiento de poderes en el territorio.

## INTRODUCTION

There is a great challenge in equitable access to quality palliative care around the world. When one thinks of vulnerated populations, those formed by individuals inserted in contexts with unfavorable conditions for life^(1)^, limitations increase, whether due to different types of discrimination, which label and separate human beings, or due to gigantic economic inequality that prevails around the world.

Given the characteristics of different life-threatening conditions, sometimes people do not always require care that requires hospitalization, but need certain care to be provided by trained health professionals in their homes. Volunteer work in several countries, over the last few years, has been highlighted as a strategy that helps to minimize discrepancies in access to health and dignified and quality care when we think about diseases that threaten continuity of life^(2,3)^.

In Brazil, there are still few organized actions that bring together active and targeted volunteering for assistance and assistance to people in need of palliative care. It is urgent in the country to implement educational, managerial, organizational and assistance actions that contemplate and ensure the dignity of people who need such care. In this context, we brought university extension as an action that can enable projects that enable the creation of a praxis linked to academic knowledge and that contributes to a transforming relationship between the university and society.

Brazilian public universities, since the last century, add university extension as part of their scope of work, formalizing the triad “teaching, research and extension”, and have their inseparability as a basic guiding principle of their operation. The most recent idea of extension consists of commitment to the production of knowledge in order to collaborate for social transformation, understanding society as an active agent, with valid knowledge, life and experiences^(4)^.

Motivated by the demand for care and challenges in the Health Care Network (RAS – *Rede de Atenção à Saúde*) in vulnerated communities and considering the fragility that health services have in welcoming patients and offering palliative care^(5)^, Alexandre Ernesto Silva, nurse and professor at the *Universidade Federal de São João del-Rei*, in Minas Gerais, since 2008, in its *lato* and *stricto sensu* training process, it began providing home care to patients with palliative care needs in Vidigal and Rocinha slums, in the city of Rio de Janeiro, Brazil, with the support of community members voluntarily. Soon there was a growing demand from the population for this care, requiring the structuring and expansion of these services.

In August 2019, when they met at the I Congress of Palliative Care in Rio de Janeiro, other professionals specializing in palliative care joined this initiative, among them two nurse professors at the *Universidade Federal do Rio de Janeiro*, Maria Gefé da Rosa Mesquita and Liana Amorim Corrêa Trotte, and a physician from the municipal health network, Lívia Pereira Coelho, who opted to build a university extension project with the intention of implementing a Compassionate Community in the territories of Vidigal and Rocinha.

The term “Compassionate Community” is not new. The first conception of a movement/action of what would later be called Compassionate Communities came about through the work of a professor at La Trobe University (Australia) in conjunction with the Victorian Department of Human Services, and started as action by the university’s School of Public Health. At this first moment, the intention was to create strategies for university-based palliative care promotion, which would generate incentives for expanding palliative care promotion in public health in Australia^(6)^.

This initiative aimed to demonstrate to the university and society that palliative care promotion also constituted health promotion care, contributing to the demystification of the idea that palliative care was only related to secondary and tertiary care and the end-of-life process^(6)^.

Derived from the World Health Organization’s concept of “Healthy Cities” or “Healthy Communities”, Compassionate Communities are based on the idea that health is more than the mere absence of disease and encourage ordinary people everywhere to adopt an understanding that health is everyone’s responsibility and not just health services and professionals. Such conceptions were gradually defended in the world, having as a milestone the Declaration of Alma Ata, which inspired the paradigm shift from hospital-centered and disease-focused models to collective models that paid attention to preventive aspects and health promotion. Throughout the late 1990s and early 2000s, palliative care professionals added the aforementioned concepts and introduced the idea of people thinking about the dying process as a step in life^(7)^.

In our country, the Brazilian Federal Constitution of 1988^(8)^ defined, in Article 196, that health is a right of all and a State’s duty, guaranteed through social and economic policies aimed at reducing the risk of disease and other health problems and universal and equal access to actions and services for its promotion, protection and recovery. However, it is believed that popular involvement is fundamental for the effective exercise of democracy and guaranteeing access to established rights. Dialogue between the population and the State must take place on a regular basis and can be carried out through social organizations and other forms of associations that represent groups or communities and act as support for achieving desirable goals.

Compassionate Communities can operate as part of a broader health approach to support people with life-threatening conditions, their families and caregivers. Some examples^(3,7,9)^ adopted so far generally involve the use of community development approaches to raise awareness about dying and build people’s capacity in the community to care for each other.

Based on this reflection and the difficulties faced in our reality as palliative health professionals working at federal public universities and the Brazilian Health System (SUS – *Sistema Único de Saúde*), this study aims to describe the implementation of a Compassionate Community in Rocinha and Vidigal slums, located in the city of Rio de Janeiro.

## METHODS

### Study Design

This is an experience report on the implementation of university extension project entitled “*Comunidade Compassiva: uma proposta de engajamento social para o fortalecimento dos cuidados paliativos*”, carried out in two slums in the city of Rio de Janeiro, RJ, Brazil, based on the conceptual bases published by the World Health Organization and by researchers Allan Kellehear and Suresh Kumar^(2,6,9)^. It was developed supported by the guidelines of university extension as an academic practice, as an interdisciplinary and transdisciplinary methodology and as a systematic dialogic interaction between the university and society.

**Figure 1.**
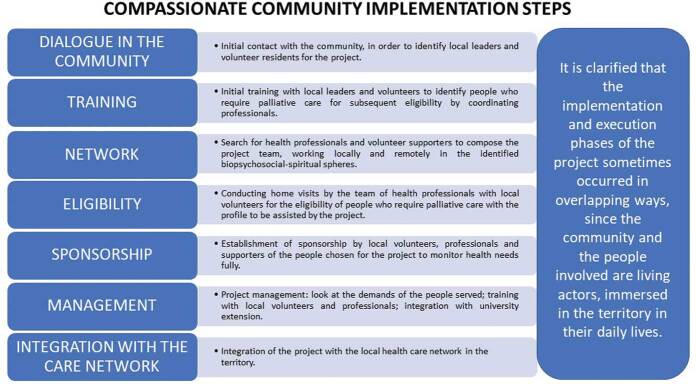
Interrelated steps for project implementation.

The implementation of this project took place through seven interrelated steps, as shown in Figure 1.

Given the lively nature of the meetings and actors in the process of implementing a Compassionate Community, there was great wealth in the meetings, which allowed promoting comprehensive health practices and the gradual construction of networks in moments with more power than in others, as that barriers that promote and hinder access, bonding and co- responsibility of those involved in palliative care and health care in the territory were identified.

### Place

The present study was carried out in the communities of Rocinha and Vidigal, located in southern Rio de Janeiro, RJ, Brazil. These locations constitute subnormal urban clusters, popularly known as favelas. Although these territories are not composed homogeneously in all regions of Brazil, they have some characteristics in common, such as the predominance of a low-income population that often sustains itself with informal jobs that do not generate social security contributions, which weakens the worker, residences built on irregular land, among others, producing difficulties in access and in the movement of people, whether or not they live in the locality.

According to the Brazilian Institute of Geography and Statistics (*Instituto Brasileiro de Geografia e Estatística*)^(10)^, subnormal urban agglomerations are housing complexes made up of at least 51 housing units, including shacks, houses, and other forms of housing, with occupation of land owned by others in the public or private sphere. In general, they present weaknesses in the coverage of essential public services^(10)^. With regard to the characteristics of urbanization, it has narrow circulation routes, irregularly shaped, plots of uneven sizes and shapes. For the most part, they are not regulated by public bodies^(10)^.

Rocinha is considered the largest slum in Brazil and one of the largest subnormal urban agglomerations in the world^(11)^. Its population is estimated at 69.356 thousand people and about 25.543 thousand households^(10)^. Vidigal has an estimated population of 12.797.000 people, comprising around 4.585.000 households^(10)^.

The present study was produced through the experience of the authors themselves, which occurred during the project implementation steps’ experiences, between 2019 and 2021. Compassionate Community implementation will be narrated through the description of activities, which will include: the eligibility of patients for palliative care; the relationship between the community, patient and health team; health education activities; home visits; volunteer actions; characteristics of territorial vulneration; clinical case discussions; and the provision of comprehensive and palliative care.

### Ethical Aspects

As this is a professional experience report, it was not necessary to be assessed by the Research Ethics Committee.

## EXPERIENCE REPORT

At the beginning of work, coordinators sought to identify people in Rocinha and Vidigal who would act as local leaders, in order to present the project, explain the objective and, thus, ask for people who had an indication to receive palliative care and others who wished to act as volunteer caregivers of these people. The gateways found in the territory were the Residents Association and the local Catholic Church. The second, being led by Franciscan priests, already had volunteers working in the health care of people.

In the quest to immerse themselves in the territory, many meetings with the residents were held with the intention of listening to the social, cultural and health context of the local people so that, in this way, relationships would be strengthened, trust would sprout and bonds would happen. In a vulnerated territory, used to the absence of the State, there are many suspicious looks and questions about what would be needed in exchange for our work. People hardly believed that the project was not linked to hidden ulterior motives, such as providing services to some party-political initiative that later resulted in asking someone for votes. It should be noted that, in moments of disbelief, we presented ourselves as professors from the Brazilian public university who sought to work executing extensionist ideals. This opened doors for us and gave credit to our real intentions, i.e., to offer palliative care to vulnerated people.

A lecture and conversations with residents about the definition of palliative care, its objectives and who would be the people recommended to receive this type of care were the first implementation initiatives. Afterwards, volunteer residents were encouraged to look for people in the neighborhood who had the characteristics presented in our meetings and who wanted to receive a visit from the team of health professionals. Thus, the new step began, consisting of home visits to people potentially eligible for palliative care.

At the beginning of this step, home visits were carried out by a small team composed of project coordinators (nurses and a physician), plus the volunteer resident, who had identified the person potentially eligible for palliative care. For eligibility, patients were assessed by the team using criteria such as: having a progressive, incurable and advanced disease; having an oscillating clinical evolution, characterized by the emergence of several crises of needs or recurrent exacerbations; having a condition that caused great emotional or social impact for them and their family; having a reserved life prognosis, with indication of predominant or exclusive palliative care; having a need for therapeutic adequacy.

For the purpose described above, the team developed its own instrument, bringing together elements already described in the literature for the assessment of eligibility for palliative care, containing biographical data of individuals, underlying disease, treatments and medications in use, symptom assessment, using the Edmonton Symptom Assessment System (ESAS) instrument, functional assessment, with support from the Karnofsky Scale (KPS), analysis of exams that the person presented to the team. Added to this were data that contemplated the local reality of the vulnerated territory, without which it would not be possible to make therapeutic decisions and propose care plans, such as housing conditions, family situation or availability of some type of caregiver, use of some income, contact with daily meals, conditions for access to some health service, among other particularities that the team deemed necessary to record in the instrument.

It should be noted that, due to the territory’s large geographic extension, many residents do not know all the regions of the slum and, consequently, the different realities that coexist in it. This finding impacted the volunteer residents, and contributed to their motivation to become even more involved with the project.

After the end of eligibility visit, when the team considered the person eligible for palliative care and with the profile to be monitored by the project, they received information that they would receive other visits from their neighbor and, monthly, from the project team. When eligibility did not happen, considering the needs identified in the assessment, the team prepared a summary of the service and guided people in the flow to access health care in the territory.

Continuing, people in palliative care are monitored by the project, strategically receiving a local “godfather/godmother”, responsible for carrying out periodic home visits, closely monitoring their “godson/goddaughter”, their needs and being the intermediary between the person and the health team. For better management and optimization of processes, patients in palliative care receive a professional tutor, who becomes responsible for handling the case, communicating directly with the local “godfather/godmother”, activating other team professionals and communicating directly with patient/family, according to identified needs.

In the beginning of Compassionate Community implementation, we were worried about the adhesion of health professionals through volunteering. This is a university extension project that does not have funding to pay staff, as we are aware of the difficulties faced by our co-workers, due to the precariousness of working conditions in the health area in our country, aggravated by the COVID-19 pandemic, which generated overload in many. However, when networking, presenting the project and requesting support, the result was remarkable: we got volunteers from all specialties in the health area, in different states of Brazil, who made themselves available to assist patients, either remotely or in person. Likewise, people from other areas and members of civil society who became aware of the Compassionate Community that was emerging in Rocinha and Vidigal began to make contact to find out how to contribute. In this way, members called “supporters” emerged, who are volunteers who identify with the project and contribute in different ways, according to each one’s availability, whether remotely or in person.

All engaged people receive guidance on volunteering, in line with the United Nations principles, i.e., our volunteers are working to achieve the Millennium Development Goals by reducing poverty and contributing to the peace, safety, health, well-being and economic, social and political development of people, thereby contributing to societies at local and national levels.

Considering that Compassionate Communities welcome volunteers, whether they are health professionals or members of civil society, another crucial aspect that the project coordination invests efforts in is education, worked through different health education methodologies, supported by evidence-based health. According to the level of knowledge of each person involved in the project, an education strategy is designed respecting the time, purpose and objective that persons had. In this way, Compassionate Communities are a constant scenario for training, conversation circles, technical visits by health professionals, undergraduate and graduate students, among other pedagogical activities that are full of knowledge and experience exchange.

We made it clear that Compassionate Communities are a support initiative that aims to expand access to palliative care for vulnerated people, which must be added to the RAS installed in the territory. There is a clear definition of the roles that each level of care must play with users in our health system, and the project aims to approach the existing care network and support it, in order to provide the user and the family in palliative care with better control of signs and symptoms as well as to gather support tools for the permanent health education of professionals involved in care.

Acting on this intention, the project coordination actively identified the network attached to the territory, composed of four Family Clinics, an Emergency Care Unit (ECU), a Psychosocial Care Center (CAPS - *Centro de Atenção Psicossocial*) and teams from the Care Program Home Care for Older Adults (PADI - *Programa de Atenção Domiciliar ao Idoso*), installed in a municipal hospital in the region, scheduling meetings for project presentation with each manager and team of these health units. At the end of the initial presentation, with the objectives of the project exposed, the health teams were presented with the users who had been eligible to receive support from the multidisciplinary team of palliative care, and it was also encouraged that the professionals of the units forward to the project users that they identified as eligible for palliative care and who could benefit from project advice.

Due to professional turnover and the presence of residents, students and other team members, semiannual meetings are held with the management and health professionals of the units. Initially, the project is presented and, subsequently, a conversation wheel is held about palliative care and the discussion of user cases.

## COMPASSIONATE NETWORK FORMATION

Implementing the Compassionate Community in Rocinha and Vidigal is a living work in health that involves relationships, creative processes, and that predominantly mobilizes structured technical knowledge and relationships between people, i.e., soft-hard and soft technologies. Little is used of the hard instrumental apparatus of hard technology, thus becoming an open space for the exploration of powers detected in it and being capable of reproduction in other territories^(12)^.

Compassionate Communities support end-of-life people and their families in many ways: help them live in their homes, connect them to services, raise end-of-life awareness, and build the capacity for empathy and compassion among people in the community. The great difference in the proposal to build a Compassionate Community is that community participation is no longer just in the sphere of operational planning, being elevated to the tactical and strategic spheres.

The care model operationalized through Compassionate Communities enables leadership incorporated into the local community, where members of these territories, in partnership with social support networks, collaborate with the provision of palliative care to people in the community who have a life-threatening condition, extending to family members and caregivers^(13)^. This care model aims to respond to the local community’ needs, usually involving people from the same geographic location, making it possible to include religious institutions, families, local organizations, neighborhoods and diverse groups of people who share similar experiences^(14)^.

Thus, the way in which Compassionate Communities develop and provide support may vary between different communities^(14)^. Likewise, the Compassionate Community developed in the Rocinha and Vidigal slums is carried out by offering palliative care to people in situations of social vulneration, promoting comprehensive care, through the prevention and management of signs and symptoms associated with physical, spiritual and psychosocial disorders, through a partnership between professional volunteer health professionals, local volunteers and external supporters.

In this context, vulnerated populations are understood to be those who experience concrete situations of social inequalities, not only being in the field of susceptibility, but actually experiencing the fragility of the State’s action^(1)^, embodied by socioeconomic and cultural disparities present in these territories. In the meantime, the context of social vulneration experienced by patients in palliative care, who live in these locations, provides a process of dying/anticipated death, which emerges from the lack of access to essential services for human life, such as health, food, housing, income, education, leisure, among others, which constitute determining and conditioning factors for the health-disease path.

The anticipation of the dying process resulting from the disparities found in these locations is known as the neologism misthanasia, an unhappy and painful way of dying, caused in slow and subtle ways as a result of certain systems and structures^(15)^, i.e., the lack of resources that infer basic human needs. The practice of this phenomenon diverges from the rights guaranteed by the Constitution of the Federative Republic of Brazil^(16)^, violating Article 1, item III, which deals with human dignity. Moreover, it opposes other fundamental rights and guarantees, such as the inviolability of the right to life, which is considered a sovereign value, which cannot be relaxed and covers everyone without distinction^(17)^.

A projection study on the future burden of severe health-related suffering found that, by 2060, around 48 million people will die each year from some form of health-related suffering^(17)^. Of these deaths, about 83% will occur in low- and middle-income countries, where there is an incipient mobilization for the provision of palliative care to the population^(18,19)^. From this perspective, these individuals, who live and die in areas of poverty, do not receive adequate relief from human suffering through palliative care or pain control. In this way, looking at these weaknesses, suffering is materialized in the cruel face of social inequalities^(19)^.

In Brazil, the precariousness in the availability of services that offer palliative care is aggravated when it comes to vulnerated communities, as there is a lack of research and government initiatives that deal with this practice in these territories. Additionally, the number of individuals who need this approach in this context is not known. Furthermore, the specific care demands that this scenario imposes have not been documented. It is imperative to encourage initiatives that provide equity, completeness and universality of access to palliative care, doctrinal principles that govern the SUS.

In this perspective, the results of this study corroborate the findings of a research that proposes a model of care that is based on the principles of social justice and equity^(20)^. In this space, the integration between specialized palliative care, general palliative care, Compassionate Communities and members of the local community is carried out for an end-of-life care approach, enabling the integration of technical-scientific knowledge and popular knowledge for a greater reach of this approach in different social contexts^(20)^.

In the Brazilian scenario, popular participation in the process of elaboration and implementation of public health policies is ensured through the Organic Health Law (LOS) 8.142/90, in which the population is guaranteed spaces to debate ideas and priority problems in the health-disease context^(21)^. In this perspective, the care developed by the local population through the Compassionate Community shows that it is a collective responsibility to take care of each other, minimizing suffering and promoting individuals’ quality of life in life-threatening conditions^(6)^, facing a health service that often does not put into practice the principle of universality.

Moreover, it is worth highlighting the role of nursing in the planning and implementation of this Compassionate Community, which, in Brazil, was an initiative led by nurses. Palliative nursing is anchored in values and skills, such as presence, listening, observation skills, assertive communication, person-centered care, empathy, compassion, among others, which make such professionals develop strategies and relational skills that make them stand out among patients, family members, staff and the community^(22)^. Taking care of a person in palliative care keeps the flame of care burning in nurses, whether through the sensitive art of a touch, silence or presence at an opportune moment, through clinical competence, by developing diagnostic reasoning to manage a certain symptom that is exacerbated.

When entering Rocinha and Vidigal to develop the Compassionate Community, our attentive eyes are flooded with stimuli for the production of care that is much greater than the mere absence of illness. By going through the alleys and their physical structures and passing by residents and local volunteers with their stories that are told to us and, finally, reaching users in palliative care and their families in their homes in search of support, we thought of strategies that can produce more health, reduce harm and promote well-being, whether in the environment’s sphere, with a view to promoting sustainability, or in the social sphere, to combat inequalities, or in the sphere of education, to empower people and expand their possibilities in society. Developing a Compassionate Community highlights our view of seeing men as social beings who live collectively and need others of their kind during the course of their lives, reinforcing social commitment and solidarity as important tools today.

It is worth mentioning the difficulties encountered along the way, which were the COVID-19 pandemic in 2020, which made us replan strategies and postpone activities, the lack of initial funding to meet some identified needs with users and professional turnover in the territory’s care network, which caused a delay in the formation of bonds and partnership with the project.

As limitations of this study, it is observed that the reflections presented here emerge from the experiences of the implementation phases of a university extension project from the perspective of three professors and a physician from the local public health network, who are part of the project management. Thus, the experiences of other employees, such as volunteer professionals, local volunteer residents and users and family members participating in the project, were not identified.

As contributions to health and nursing, this initiative stood out for being a university extension project led by nurses who seek cooperation and dissemination of scientific, technological and cultural knowledge through the articulation between theoretical knowledge and its community application, privileging the space for the production of scientific knowledge through research, professional training and the possibility of social transformation, understanding society as an active agent, with valid knowledge, lives and experiences.

## CONCLUSIONS

With this experience report, we describe the Compassionate Community implementation in Rocinha and Vidigal slums, in the municipality of Rio de Janeiro, in order to expand access to palliative care for vulnerated people in territories with difficult entry and mobility. It can be observed that providing and continuing health care, with emphasis on palliative care, for the people who live in that place becomes a major challenge for the local health network. Initiatives such as the project described can contribute as an additional resource, in order to minimize barriers that hinder access to health, as living support networks emerge that can directly impact cases.

A Compassionate Community, through its volunteers, professionals and supporters, provides mobility to people in palliative care through meetings and care actions shared by all. In line with this assertion, related initiatives in the world, such as the 2022, of the Worldwide Hospice Palliative Care Alliance (WHPCA), whose theme was “Healing hearts and communities”, which worked on the collective grief that the world experiences post-pandemic, and was involved in conflicts between nations, and the 2023, of Public Health Palliative Care International (PHPCI), whose theme is “Compassionate Communities: Together for Palliative Care”, stand out. Compassion as the driving force of palliative care is able to contribute to building relationships of trust and comfort, in which patients, family members and professionals reinvent health production according to the leading actor’s needs.
